# *In Virus Veritas* Lockdown and Happiness Under COVID-19

**DOI:** 10.1007/s11205-022-02974-x

**Published:** 2022-08-01

**Authors:** Salvatore Bimonte, Luigi Bosco, Arsenio Stabile

**Affiliations:** 1 Department of Economics and Statistics, University of Siena, Siena, Italy; 2 Department of Business and Law, University of Siena, Siena, Italy

**Keywords:** Happiness, Social capital, COVID-19, Lockdown, I30, I31

## Abstract

It is widely accepted that individual happiness is not, or not solely, related to material possessions, at least once basic needs are fulfilled. It has been demonstrated that interpersonal relationships and social capital matter too, and people whose values are more centred on material possessions have a greater probability of being less happy. Is this still true during the COVID-19 pandemic, when interpersonal relations, health and economic security are threatened and feelings of insecurity emerge? This is the issue that we address in this paper. We exploited the unique natural situation of the pandemic and lockdown in Italy to investigate the relationship between happiness and relational and material goods. Data collected by questionnaire during the lockdown suggests that the main direct effect of the pandemic on the happiness of respondents was related to the effect of the pandemic and lockdown on interpersonal relationships. Those who declared that COVID and lockdown had jeopardized their interpersonal relationships were significantly less likely to report higher levels of happiness, especially when controlling for other personal and contextual covariates. An important gender, religious and town size effect also emerged. Moreover, relational goods and good health were considered to be the most important determinants of happiness, though people were not so worried about their own health.

## Introduction

Since antiquity, philosophers have reflected on what contributes to a better life and makes people happy. In recent decades, empirical studies on this issue have flourished in a variety of disciplines (for a review, see among others Dolan et al., 2008, and Powdthavee 2007, or the monograph Frey 2018). However, while understanding which factors impinge on subjective well-being is of utmost importance in general, it becomes even more intriguing in situations where some major determinants of happiness are jeopardised by widespread contingent factors. This surely happened in 2020 due to the COVID-pandemic.

The whole World Happiness Report 2021 focused on the effects of COVID-19 on the structure and quality of people’s lives (Helliwell et al., 2021). According to it, during this time emotions changed more than did life satisfaction. The aim of the present paper was to exploit the COVID-19 pandemic as a “natural experiment” to investigate individual happiness and the effects of some period-specific determinants. Moreover, unlike the WHR 2021, we carried out the research during the lock-down period and, instead of analysing satisfaction with aspects of individual life, we investigated on the factors people believe contribute most to individual well-being. This was done on the assumption that under an extraordinary and dramatic event reference values may change or, at least, what people really care about may come to the fore more spontaneously.

A Latin saying, *in vino veritas* (in wine lies the truth), suggests that persons under the influence of alcohol are more likely to express their true views and desires. Alcohol loosens moral restraints, enabling people to express what they really think and feel. We rephrased the saying as *in virus veritas*, under the assumption that COVID may have a similar effect on people’s feelings, being an extraordinary event that has dramatically changed the way our lives are organized and conducted. What people really care about may emerge more spontaneously than at other times, or at least, COVID may change the relative importance of things (scale of values) and the way they relate to individual happiness. Of course, we are not suggesting that studies conducted in “normal” times are deceiving and misleading. Our main point is that there are special moments in life when people see their attitudes and preferences in a different and extraordinary way. By observing agents in such situations, we may capture information that is otherwise not available. The pandemic gave us this opportunity. The evidence we tried to obtain during this unique natural experiment is also unique, as well as complementary (not necessarily substitutive) of evidence captured in normal and ordinary times.

The COVID-19 pandemic is a unique event, unlike anything we have seen in the recent past. Its uniqueness lies not only in the dimensions of the viral attack but also in the response to the threat by policy makers in various countries. The lockdown measures implemented in some countries forced millions of people to remain at home for weeks and in some cases, as in Italy, for about two mouths. It goes without saying that the Italian experience was also unique in the violence of the epidemic, the human, social and economic costs and the time spent in the lockdown. It had and continues to have many dramatic effects in terms of deaths and economic costs, all of which have yet to emerge. However, it certainly (and even more) modified people’s habits and threatened their lives and relationships, especially in the lockdown period (Helliwell et al., 2021).

This is different from what Kahneman (2011) defines as a focusing illusion. In his view, contextual factors may cause judgment bias. Instead of assessing their lives as a whole (target question), people focus on their contingent affective state (heuristic question). Rather, it is more in line with what Thaler and Sunstein (2008) sustain regarding arousal state, which varies in time, giving rise to dynamically inconsistent appraisals. In a cold state (in our case “COVID-off”), individuals do not fully appraise the importance of certain aspects of their lives. In a hot (aroused) state (in our case “COVID-19”), they appraise them differently, due to the effects of “arousal” on their mood or value system. In the language of economics, one can say that actual experience modifies agents’ perceptions, changing their indifference curves and thus their appraisal (Bimonte & Punzo, 2016). Whether or not this modification endures is another question.

Empirical studies on life-evaluation have identified a number of variables explaining changes in individual happiness. The variables can be grouped in three main categories: economic (such as income, employment status), social (education, marital status, social capital) and health-related (physical and mental) (DeLeire and Kalil 2010; Dolan, Peasgood, and White 2008; Easterlin 2003; Frey, 2018; Frey, Stutzer, and Easterlin 2002; Helliwell, Layard, and Sachs 2017; Rojas 2011). There is now consolidated empirical evidence showing that once basic needs are met, material possessions do not contribute significantly to individual happiness (DeLeire and Kalil 2010; Helliwell 2003; Kasser, 2002). It has been demonstrated that relational goods and social capital also matter (Bruni and Stanca 2008; Diener & Seligman 2009), and people whose values are centred more on material possessions or income aspiration have a greater probability of being less happy (Kasser, 2002; Killingsworth and Gilbert 2010).

The COVID-19 pandemic and the policy response (lock-down) provide a kind of counterfactual situation. The latter concerns how things would have been under a particular/hypothetical circumstance. In our specific case, the (rhetorical) question is: what would happen to individual happiness if people were faced with a threat to their economic, social and relational situation? Would relational aspects still prevail over material possessions in determining individual happiness?

However, since such a situation happened in 2020 and the pandemic did indeed affect people’s economic, social and relational situation, undermining their feeling of security, it offered an “opportunity” to address our question. Under the “*in virus veritas*” assumption, we tested whether dependence of happiness on material aspects persisted under lockdown or whether sociability turned out to be an important determinant of happiness. We addressed the following questions:

Q1. In the dramatic lockdown period, income, health and interpersonal relationships were threatened. How did this threat affect reported happiness?

Q2. Under these threats, were social capital and relational goods as opposed to more material aspects still considered important determinants of happiness?

Q3. Did the effect of lock-down on happiness depend on individual characteristics?

To address these questions, we carried out a web-survey during the COVID-19 lockdown period. Ordinal Logit Regression (OLR) was used to detect whether (and how) perceived or actual COVID impacts, respondents’ profiles and factors considered important for a happy life related to individual happiness. As for the latter aspect, unlike other papers that investigate people’s satisfaction with aspects of their lives, we investigated their opinion of what matters for a happy life, while assessing their happiness and self-reported impact of the pandemic on income, personal relations and health. Our outcomes suggest that under lockdown, the main direct effect on happiness was through the effect on interpersonal relationships. Subjects who reported that COVID and lockdown had threatened their interpersonal relationships were significantly less likely to report higher levels of happiness. This result is always true, irrespective of the regression model used. Its strength and significance increase when controlling for other important covariates. Moreover, social capital and social interpersonal relationships also emerged as important determinants of happiness. An important gender effect emerged as well. However, considering the sampling procedure, imposed by time constraint and the difficulty of administering questionnaires during lockdown, results has to be taken with caution.

## Method

### Sampling and procedure

The questionnaire was administered by online survey using Google Forms. Participants were recruited mainly using Facebook and WhatsApp. People were invited to participate in a survey by connecting to a web link. Aware of all its limitations, this technique was employed for the following practical reasons: lockdown made it impossible to conduct face-to-face interviews; there were insufficient time (lockdown period) and resources to build a more accurate survey^1^. Before the starting the interview, respondents were informed of the aim of the study and its non-commercial purpose. They were also told that the survey was confidential and anonymous, and that the results would only be released in aggregate form.

According to a standard widespread procedure (Frey & Stutzer, 2002; Veenhoven, 2007)^2^, in the first question respondents were invited to answer the following question “All things considered, how happy are you with your life as a whole?’ and to assess their happiness on a 10-point Likert scale (1 unhappy, 10 very happy). The rest of the questionnaire was organized as follows. The first section was designed to investigate respondents’ opinions on what specific life domains make people happy. Aspects of life included in this section have been widely reported in the literature, e.g. economic situation, work, personal relations, family, prestige, and so on (Frey & Stutzer, 2002, Di Tella and MacCulloch, 2010; Layard 2005; Rojas, 2019). The second section was designed to detect respondents’ opinions on the aspects of societal life and contextual factors that contribute most to individual happiness. As before, we considered aspects that have been widely investigated in the literature, e.g. inequality, pollution, security, immigration and so on (see for example Alesina et al., 2004; Rojas, 2011). In both cases, respondents were invited to answer the following question “According to you, how important are these features in the pursuit of individual well-being?” and rate them on a 10-point Likert scale, ranging from 1 “extremely unimportant” to 10 “extremely important”.^3^

The third section contained questions aimed at recording the socio-demographic characteristics of respondents. Finally, some specific questions for the ongoing period were included. The first aimed to assess whether respondents were worried about their health. Then they were asked whether there were cases of COVID-19 among their relatives and friends and to what extent their personal relations and economic situation were harmed by the pandemic.

### Sample characteristics and descriptive analysis

A total of 401 subjects, 166 males (41.4%) and 235 females (58.6%), mean age 37.6 years, was recruited. Table 1 shows the main characteristics of the sample. The majority were from the central regions of Italy (51%), living mainly in small and medium-sized towns (median 34,000). Most respondents declared that they did not hold religious beliefs (33%) or were agnostic (19.9%). About 60% had a Bachelor’s degree or higher.

**Table 1:** Main characteristics of the sample (descriptive statistics)

Variables		*Mean (SD)*	*Median*	*Value %*
**Gender**	*Male*			41.4%
	*Female*			58.6%
**Age**		37.6 (16.35)	33	
**Region of Italy**	*North* *Center* *South and islands*			18.8% 50.9% 30.3%
**Hometown population**		182,128 (656,751)	34,000	
**Religious faith**	*Believer*			47.1%
	*Non-believer/agnostic*			52.9%
**Education**	*Lower secondary*			2.3%
	*Upper secondary*			37.8%
	*University (Bachelor)*			24.9%
	*Master*			32.2%
	*PhD*			2.8
**Impact of COVID-19 on social life**	*A lot*			36.9%
	*Somewhat*			36.2%
	*Little*			20.4%
	*Almost nothing*			6.5%
**Impact of COVID-19 on income**	*A lot*			11.7%
	*Somewhat*			26.4%
	*Little*			35.2%
	*Almost nothing*			26.7%

Almost three-quarters of respondents declared that COVID-19 harmed (a lot or somewhat) their social relations and about 38% stated that it reduced their income. Surprisingly, our sample perceived a greater effect of COVID and lockdown on their human relationships than on their economic situation/income. As we know, the economic effect of COVID in Italy was huge. Nonetheless, they perceived a greater effect of lockdown on their relational life. This outcome may depend on different factors, first of all, the timing profile: while the effect of the pandemic and lockdown on relational life was immediate, the income and economic effect of the disease will take time to manifest.

**Fig. 1:**
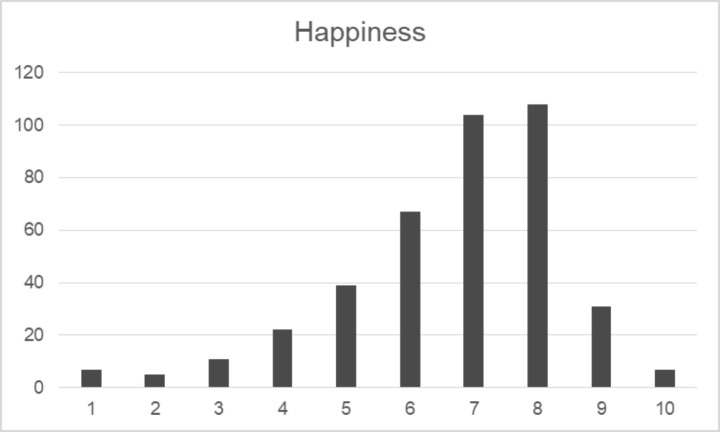
Distribution of answers

Table 2 shows the main descriptive statistics for perceived happiness and respondents’ appraisal of various factors (life domains and aspects of societal life) influencing it. People were not asked to rate their satisfaction with aspects of their lives or contextual factors, but to rate the importance of the various items in the pursuit of individual happiness. Our sample had a median happiness score of 7, with moderate left skewness and kurtosis (Table 2; Fig. 1).^4^ As for aspects of their own lives, besides good health, people appraised more “immaterial” factors, all regarding relational aspects (family, love, friendship), than “material” factors, such as high income and social status. With some differences, they were concerned about societal and institutional aspects, rating all except “Immigration” and “Loss of national identity” as very important in the pursuit of happiness. Finally, people were worried about the coronavirus, but less than we expected (median 7).

**Table 2:** Descriptive statistics of sample (n = 401)

Single items	*Median class*	*Skewness*	*Kurtosis*
Happiness	7	-0.9575	4.049
***Factors contributing to happiness***			
***Aspects of personal life***			
Job stability	8	-1.415	7.021
Social prestige/career success	7	-0.7111	3.181
Serene family life	9	-2.25	11.03
Happy love life	9	-1.561	6.391
High income	7	-0.8775	3.992
Good health	10	-2.672	12.47
Good personal relations	9	-1.425	6.748
Spare time	8	-1.079	5.022
***Institutional and societal aspects***			
Public health system	8	-0.9471	4.75
Pollution	8	-1.02	4.202
High quality public services	8	-0.8804	4.054
Threatened sense of community	8	-0.9456	3.994
Immigration	5	-0.113	2.196
Corruption	8	-1.195	4.244
Crime	8	-1.057	4.044
Organised crime (mafia)	8	-1.403	4.971
Inequality	8	-1.469	5.486
Tax evasion	8	-1.206	4.313
Loss of national identity	7	-0.6227	2.663
**In this period, are you worried about your health?**	**7**	-0.7262	3.2

### Model and regression analysis

In our study we assumed that the model has the following underlying relation:

1 $${Y}_{i}={\beta }_{1}+{\beta }_{2}{A}_{i}+{\beta }_{3}{P}_{i}+{\beta }_{4}{I}_{i}+{\mu }_{i}$$

where ${Y}_{i}$ is a measure of subjective well-being of individual *i*; *βs* are parameters; *Ai* is a variable expressing attitudes and believes ; ${P}_{i}$ is a vector of personal and contextual characteristics (sex, age, education, faith, hometown population, concern for health); ${I}_{i}$ is the impact of COVID on income and social relations of individual *i* (income-covid and social-covid), and ${\mu }_{i}$ captures the remaining unobserved variation, assumed to be unrelated to the explanatory variables.

Considering the characteristics of our response variable and the factors considered predictor variables in the survey, we ran a regression model for categorical variables, i.e. an Ordinal Logit. The use of models such as the OL is appropriate whenever the variable of interest is ordinal (McKelvey and Zavoina 1975; Winship and Mare 1984). This model is defined by a set of *N-1* equations where the logit of cumulative probability of a response variable *Y*_*i*_ with *N* categories is assumed to be related to the covariates *X*_*i*_ whose regression coefficients *β*_*i*_ remain constant across response categories, i.e. Ho:=*β*_*1i*_ *= β*_*2i*_ *= β*_*3i*_*=…= β*_*(n−1)i*_ *= β*_*i*_. This is known as the parallel regression assumption. If the latter is satisfied, the following model can be estimated:

2 $$\text{Pr}\left({Y}_{i}<j|{X}_{i}\right)=\frac{\text{e}\text{x}\text{p}({u}_{j}-\varvec{\upbeta }{\mathbf{X}}_{\mathbf{i}})}{1+\text{e}\text{x}\text{p}({u}_{j}-\varvec{\upbeta }{\mathbf{X}}_{\text{i}})}$$

It estimates the coefficients that maximize the joint probability of the observed values of *Y*_*i*_, conditional on observed *X*_*i*_. The coefficients estimate the effect of a variation in the explanatory variables on the probability of getting better scores in the response variable. The impact of a one-unit increase (reduction) in a given predictor depends on its starting value and the values of the other predictors. However, in general, one can say that a positive (negative) coefficient means that an increase in the predictor leads to an increase (reduction) in the likelihood of obtaining a higher score for the dependent variable, quantified by the coefficient (Long and Freese 2006).

Considering the sample size and the distribution of answers, we collapsed the original 10-point responses variable ${Y}_{i}$ into three categories: 1–5, 6–7, 8–10, corresponding to unhappy, happy and very happy (Helliwell & Huang, 2013; Schwarze et al., 2011) in order to construct categorical indicators with sufficient observations and make results more easily interpretable and reliable (Table 3).

**Table 3:** Distribution of answers

Happiness	Freq.	%	Cum.	
1= | <6	84	20.95	20.95	
2= | 6–7	171	42.64	63.59	
3= | >7	146	36.41	100.00	
Total	401	100		

In our model, attitudes were defined according to the importance that respondents assigned to the eight personal life variables and the eleven institutional and societal variables as determinants of individual happiness (Table 2). The dataset evidenced correlation between many of these variables. Therefore, Principal Component Analysis was carried out in order to reduce dimensionality of our dataset. In conducting it, we considered only principal components that had an eigenvalue greater than one (see Table 1A1 and 1Ab in the appendix).^5^ The resulting five principal components were the variables that we used as regressors in the model.^6^

The first principal component grouped corruption, tax evasion, petty crime, organized crime (mafia) and income inequality. Since these items can be seen as important elements of social capital, we named it *Social Capital*. The second aggregated good health, sound family situation, happy sentimental life and good friendships relations, and we named it *Health & relationships*. The third comprised quality of health services, quality of public services and pollution; we called it *Public goods*. The fourth aggregated stable working position, high income and social prestige, and we called it *Materialism*. The fifth grouped immigration and loss of national identity, and we called it *Populism*.^7^

## Discussion of results

Table 4 summarizes the main estimates. The four columns represent different specifications of the model. Specification I is the model of Eq. (1) while the others progressively exclude variables that turned out to be non-significant in the first specification. Diagnostic tests confirmed the robustness of the results. The first three rows show the effect of what we considered the main direct drivers of happiness in our model. To make things simpler, we collapsed the original four-level survey responses (see Table 1) of these three variables into two levels (dummy variables) aggregating “a lot” with “somewhat” and “little” with “almost nothing”. The results show that of these three direct drivers of happiness during the pandemic and lockdown, only relational-covid appeared to have a significant (negative) effect on the probability of a higher level of happiness. This is so, irrespective of the regression model used, and its significance improves when controlling for other covariates.^8^ Although they had the expected sign, income-covid and health-covid turned out not to be significant. It is worth recalling that, unlike what normally done in other studies, these variables were evaluated in term of impact, i.e. variation induced by COVID.

The effect of relational-covid was remarkably great. Persons who reported that COVID affected their relational life a lot or to some extent had a 45%^9^ lower probability to report higher levels of happiness. Income-covid had the expected negative sign and had less impact than relational-covid (18%), but was not statistically significant. These results were also confirmed by the other models. This may be partly due to the fact that the social lockdown and consequent difficulty in spending money made money temporarily less useful than usual and therefore less desirable. If it is not money itself that affects happiness but the goods and the services that money buys, the obvious difficulty of purchasing goods during lockdown may have reduced the influence of income on happiness. The same was true of health-covid. Fear of COVID was quite common in our population but did not influence the probability of being very happy.

**Table 4:** Regression results

Happiness		(1)	(2)	(3)	(4)
Relational-covid	-0.593***		-0.603***	-0.578***	-0.566***
	(-2.732)		(-2.843)	(-2.705)	(-2.644)
Income-covid	-0.202		-0.217	-0.252	
	(-0.942)		(-1.035)	(-1.237)	
Health-covid	0.0625				
	(0.294)				
Education	0.318				
	(1.550)				
Hometown population	-0.437**		-0.437**	-0.439**	-0.413**
	(-2.192)		(-2.202)	(-2.202)	(-2.122)
Age	-0.00279				
	(-0.406)				
Gender	-0.507**		-0.488**	-0.429**	-0.446**
	(-2.489)		(-2.418)	(-2.126)	(-2.210)
Gender# Health and relat.	-0.385***		-0.367***	-0.319**	-0.334**
	(-2.759)		(-2.647)	(-2.387)	(-2.477)
Religious faith	0.730***		0.691***	0.685***	0.697***
	(3.534)		(3.524)	(3.525)	(3.578)
Social capital	0.134**		0.142***	0.140***	0.136***
	(2.399)		(2.608)	(2.638)	(2.584)
Health and relationships	0.393***		0.388***	0.357***	0.362***
	(3.644)		(3.722)	(3.529)	(3.555)
Public goods	-0.0975		-0.105		
	(-1.056)		(-1.157)		
Materialism	0.174		0.151		
	(1.566)		(1.390)		
Populism	0.132		0.139		
	(1.139)		(1.205)		
Constant cut1	-2.065***		-2.202***	-2.155***	-2.036***
	(-4.809)		(-7.126)	(-6.895)	(-7.062)
Constant cut2	0.0547		-0.0926	-0.0711	0.0406
	(0.129)		(-0.315)	(-0.241)	(0.148)
Observations	397		397	397	397
Wald	47.12		45.01	39.20	37.46
Prob > Chi2	1.84e-05		4.84e-06	4.51e-06	3.84e-06
PseudR2	0.0657		0.0625	0.0549	0.0530
LL	-391.8		-393.2	-396.4	-397.2
*Robust z-statistics in parentheses* - *** p < 0.01, ** p < 0.05, * p < 0.1					

With regard to the role of personal aspects, age and education turned out to be not statistically significant. This is not surprising. The happiness literature is unclear about the link between age and subjective well-being, nor does it show any clear-cut conclusions on the link between education and subjective well-being. Some empirical studies have found a positive effect of education on happiness (Di Tella et al., 2001, Stevenson and Wolfers, 2008, Cuñado and de Gracia, 2012), while others have shown a non-significant (Inglehart & Klingemann, 2000) or even negative effect (Clark and Oswald 1996). While a direct positive effect of education on SWB is understandable, being evidence of “self-confidence” or “self-esteem”, the negative effect has been explained by the fact that a higher level of education may increase the misalignment between expectations and fulfilment. This was suggested by a paper on highly educated Italians (Ruiu and Ruiu 2019).

In our case, results may depend on the characteristics of the sample, where about 60% of respondents had at least a bachelor’s degree. The variable had a low variability and was skewed on the right. Education may be used as a proxy for employment status and hence income. Both are usually associated positively with happiness. Due to the percentages mentioned just now, this effect was not caught by our sample.

Interestingly, the effect of religious faith appeared to be positive and significant, i.e. believing increases the probability of being happy. Those who defined themselves as religious had more than twice the probability (108%) of reporting a higher level of happiness than those who defined themselves as not religious. The question about religious faith was very broad (Would you define yourself as a believer?) for two main reasons: first to lighten the questionnaire and obtain more reliable answers; second, we were not interested in differentiating faiths or observation. Our idea was to capture an internal sense of religious faith and to determine whether it can affect happiness, especially in unusual times like the pandemic. The variable scored one if the answer was positive and zero otherwise (i.e. no or do not know).

This result is in line with other studies that found positive relationships between subjective well-being (SWB) and religious faith in various populations, irrespective of religion (see Minkov et al., 2020 and references therein). The role of participation in groups and networks with religious inspiration was stressed by Lim and Putnam (2010). Since our questionnaire was administered during lockdown, participation was prohibited and this effect should be absent or very small. In our case, we can guess that feeling part of a believer community, in most cases the Catholic Church, may have a positive impact on happiness, especially in anxious and uncertain times, such as the pandemic. Moreover, faith is somehow a form of certainty and self-assurance which can improve people’s self-confidence. In a period such as lockdown, this can have a positive effect on happiness.

Interestingly enough, hometown population also had a significant coefficient. In the regression we constructed a dummy variable equal to zero if the population was less than 10,000, and one otherwise. The coefficient was negative and significant: living in a small village contributed to individual well-being. Those who lived in larger towns had 35% less probability of reporting a higher level of happiness.

The relationship between happiness and town size, or happiness in rural versus urban areas, has often been studied, without reaching clear-cut conclusions (Itaba, 2016). Some studies found higher levels of SWB in rural areas (Shucksmith et al., 2009, Knight and Gunatilaka, 2010, Davern and Chen, 2010); others found the reverse relationship (Millward and Spinney, 2013), while in some cases no sharp disparity emerged (Mookherjee, 1992; Best et al., 2000). This relation may depend on the stage of a country’s development: in less-developed countries happiness is considerably greater in urban settings, but this urban–rural differential tends to disappear in developed countries (Easterlin et al., 2011).

Using a very large dataset for community-level life satisfaction in Canada, Helliwell and co-authors recently concluded that “life is significantly less happy in urban areas” (Helliwell et al., 2018).^10^ Our results seem in line with Helliwell’s. On theoretical grounds, living in a small village during a pandemic can have negative and positive side effects. First of all, living further from large hospitals, where the quality of healthcare can be perceived as higher, can increase anxiety. Some on-line services common in big cities are not available in small towns (e.g. home delivery of food). When people are forced to stay at home, the size of the house may be more important than the size of the town. Houses are generally larger in small towns due to lower housing prices, and more likely have gardens and surrounding countryside where people can potter or walk without breaking the lock-down rules or infecting/being infected by others. Finally, remaining at home in a small town engenders less feeling of isolation than in a city (Mouratidis and Yiannakou 2022). There is more feeling of community and people help each other. Neighbourhood relationships are real and tangible. “Mr. Smith” is not just a name on a door seen from the elevator, but a person in flesh and blood, known to the townspeople. Our result suggests that the positive elements are more important than the negative ones, and is consistent with the results of other studies, since many positive effects of living in a small village are relational. As evidenced in the last World Happiness Report, trust and the possibility to count on others are very important aspects for life evaluations, especially in the face of crises (Helliwell et al., 2021).

Another interesting finding was the significant negative coefficient for gender. In Italy, women seem to be less happy than men (ISTAT, 2020). Our results suggest that this effect is strong. Indeed, according to our computation, being female reduced the probability of being very happier by about 40%. The reasons invoked to explain the lower level of happiness of women in Italy may also explain the lower probability of happiness in our particular sample, namely the double burden of work of wives who work at home and outside (see Zoch et al., 2021). Although we do not have data on this aspect, it is likely that most of the burden of the lockdown was carried by wives. During lockdown schools were closed and children stayed home, dramatically increasing the time dedicated to childcare. If the division of housework is unfair and the bulk of it falls on wives, it is not surprising that lockdown reduced the probability of female happiness.

## Happiness and personal beliefs

In this last section we concentrate on the relationship, if any, between happiness and individual beliefs about happiness determinants. We try to establish whether beliefs about what is really important for being happy are somehow related to reported levels of happiness. Again, we did not ask respondents about their satisfaction with different domains of their lives, but how important they believed these domains to be for individual happiness. As stated before, the questionnaire contained two groups of questions aimed at appraising respondents’ attitudes regarding the main drivers of subjective well-being. We distinguished personal and institutional/social aspects (Table 2). Then, we carried out a Principal Component Analysis to reduce the dimensionality of our dataset. It found five components that we called: social capital, health & relationships, public goods, materialism and populism.

The first point worth noting is that the regression results preserved the distinction between the social and individual dimensions that we postulated. As Table 4 shows, only social capital and health & relationships were statistically significant. In both cases the sign was positive. Social capital turned out to be an important determinant of happiness. This may appear partially counterintuitive. Italy cannot be defined as a country with robust social capital. To the contrary, corruption is high, tax evasion is dramatic and widespread, and organized crime is stronger and more pervasive than in other countries. Individuals who believe that these elements are important may therefore be expected to display a lower level of happiness, given the Italian situation. However, once again, it is worth recalling that we investigated what people believed to be important to pursue happiness rather than how satisfied they were with aspects of their own lives and environment. Our results suggest (and somehow confirm) that pro-social and less materialistic beliefs foster happiness. Believing that social capital is an important driver of subjective well-being also means believing in a cooperative idea of society and feeling that a society which faces an external enemy, such as the coronavirus, in a united and collaborative way has a greater chance of success.

This result was consistent with and reinforced by our findings with respect to health & relationships. Relational goods are local public goods which are simultaneously consumed and produced (Gui and Sugden 2005). Examples of relational goods are affective relationships such as love, family relationships, friendship, neighbourhood connections, and many other kinds of social interactions. Our findings suggest that people who say that sound health and relationships are an important determinant of happiness are more likely to report a higher level of happiness, although this applies with different strength to women and men. In fact, the interaction term between “Gender” and “Health and relationships” turned out to be negative and significant.

In a period when health and relations are widely threatened, this may appear counterintuitive, especially if we consider that subjects who reported a worsening of their interpersonal relationships due to lockdown also showed a lower level of happiness. We would expect persons who give relatively more importance to relational goods to have a lower probability of reporting a higher level of happiness. Deeper analysis shows that this result is consistent, if we distinguish between “attitudes” and “COVID impacts”.

Being pro-social and pro-relational is good for individual well-being. According to our assumption “in virus veritas”, this is particularly true in dramatic times, when people focus more on what makes life worth living. Moreover, those who give relatively more importance to health and relational goods are likely to organize their lives coherently. They are more likely to have a healthy life-style and to observe the rules of hygiene. They feel equal to the challenge of the virus and may therefore be happier. Similar considerations apply to relational goods. Those who believe that health and relational goods (friendships, sentimental relationships) are important drivers of well-being are likely to organize their lives accordingly. They build a rich and varied network of friendships and invest in cheerful sentimental relationships with their partners. It is therefore likely that persons who value relational goods, consume more of them than those who believe that such goods are not important. Since a positive correlation between relational goods and happiness has been demonstrated in different empirical studies (Becchetti et al., 2008, Gui and Stanca, 2010), it is legitimate to expect that those who value relational goods end up reporting higher subjective well-being. On the other hand, in line with our “counterfactual” hypothesis, because they value relational and social aspects, those who more experienced a worsening of these aspects turned out to be less happy. To say it in a sentence: the more you appreciate a good the more you suffer when is gone.

The other three principal components, public goods (quality of health services, quality of other public services and pollution), materialism (job stability, high income, social status) and populism (immigration and loss of national identity) were not significant.

As far as materialism and populism are concerned, the happiness literature suggests some interesting results. In a major meta-analysis, Dittmar et al., (2014) showed a negative association between materialism and well-being with small negative correlations between various measures of materialism and various aspects of well-being. Our definition of materialism was less rigorous than that of most other scholars, but in our sample we did not find any statistically significant relationship between the degree of materialism (as we defined it: aggregation of income, social prestige and a stable job) and the probability of reporting a higher level of happiness. Again, we can interpret this outcome as further confirmation that during a pandemic, materialist aspects of life do not have any effect on happiness or people reconsider own scales of values.

Interestingly, a recent study suggested that unhappier people were more likely to vote for populist parties (Algan et al., 2018). We did not find any statistically significant relationship between the measure of populism that we adopted and reported levels of happiness. Subjects who believed that immigration and loss of national identity were important determinants of happiness did not appear to report a different level of happiness from others. Under lockdown, issues such as immigration and loss of national identity are presumably perceived as less of a threat and therefore did not have a major effect on happiness. This may also be due to the fact that during the pandemic, the media and news were monopolised by pandemic-related issues.

## Conclusion

This study was based on a research assumption: “in virus veritas”. Paraphrasing a Latin saying, it assumed that in a hot or aroused state (here due to the threat of coronavirus infection), people are more likely to express their true views and desires and may modify their scale of values. Lockdown was an extraordinary time in Italian life. Faced with an invisible and dangerous enemy, the way people organized their lives changed dramatically. As a consequence, what they really cared about (what makes life worth living) emerged more spontaneously.

Building on this assumption, we exploited the lockdown, imposed in Italy in March 2020 in response to COVID-19, to conduct a web survey into people’s beliefs about the social and personal aspects most important in the pursuit of happiness, and to appraise individual happiness and the impact of the pandemic on some aspects of personal life (income, relationships, health). The main aim was to test whether any relationship existed between beliefs, COVID impacts and reported happiness. Our aim was to determine whether the picture was so different from those recorded in “ordinary” periods in Italy and elsewhere. In other words, we set out to discover whether the unique situation in which Italians were forced to live could give us new information on the relationship between happiness and its determinants.

Our heuristic hypothesis was that the pandemic and the policy response (lockdown) represented a counterfactual situation that allowed us to address the following rhetorical questions: what would happen to individual happiness if people were faced with a threat to their economic, social and relational situation? Would relational aspects still prevail over material possessions in determining individual happiness? Is there any relationship between people’s rescaled beliefs and reported happiness? Since such a situation actually arose in 2020, undermining people’s feeling of security, we exploited the lockdown period to address these questions.

Three main direct effects of the pandemic on happiness were postulated: an income/material effect, an interpersonal relationships effect and a health-threat effect. Our outcome suggests that only the second effect had a significant and negative effect on happiness. Of course, this does not imply that income is no longer an important determinant of happiness. It simply means that during lockdown, people felt that COVID was much more of a threat to their relational life than to their material well-being. Our interpretation of this outcome is that the viral threat and life in lockdown forced people to appreciate relational goods that may be taken for granted in normal times or underrated with respect to material goods. *In virus veritas*. As we have already mentioned, we cannot know whether this is just a short term result/effect.

The result is consistent with other aspects unveiled by our study. Religious faith, living in a small village and male gender increased the probability of reporting the highest level of happiness. The first two aspects are directly related to relational/community issues that may have mitigated the impact of the pandemic on relational goods. If this interpretation is correct, the gender effect is also coherent. Women suffered the burden of COVID more than men and their relational ties were probably more affected.

Lastly, our study also showed that people who believe that social capital and relational goods are important determinants of individual happiness tended to have a higher probability of reporting the highest level of happiness. Our results therefore tell us that the pandemic led people to appraise immaterial aspects more than material possessions, and this was a reward in terms of individual happiness. Because social capital is important for happiness, those who most experienced a deterioration in personal relations reported a lower level of happiness. The income effect, although it had the right sign, proved to be not statistically significant, as did being a materialistic type of person.

The word “*war*” was often used in the news and in political debate to describe the exceptional nature of the pandemic period. During a war, we prefer to live in a united cohesive community, rather than in a divided and highly competitive society. In other words, those who believe that social capital is important for their well-being are the same as those who believe in a strong and cohesive society.

Our main intuition was confirmed: in a period of emergency, when one’s health and that of one’s family is threatened, it stands to reason that attention shifts from material to non-material things. In a time of crisis, danger to health and uncertainty, true values emerge: *in virus veritas*. In such situations, relational goods and trust seem much more important than private and material goods. Our result suggests that the effect of the pandemic and lockdown on subjective well-being is related to their effect on social and interpersonal relationships. The other two channels we tested did not show any significant effect on happiness. This can be seen as the main result of our study. Of course, our analysis only captures immediate effects; in the long run, the income effect will presumably emerge as well.

In conclusion, our study exploited the natural experiment of lockdown to obtain interesting insights into happiness and its determinants in a unique situation. The results are intriguing and add a new perspective to the study of happiness. They somehow confirm what the World Happiness Report (2021) evidenced for the pre-lock-down period. It seems that emotions changed more than did life satisfaction.

The main shortcoming of the study was its small, non-representative statistical sample due to the time constraint and the difficulty of administering questionnaires during lockdown. This prevents any generalizability of findings. Lockdown was a (hopefully) not reproducible event that makes our analysis unique. However, considering the above mentioned shortcomings, it should be taken as such, i.e. an opportunity to address/discuss about some intriguing issues in a special environment/context, aware that, to use Corbin & Strauss (1990: 191), our results and theoretical formulation applies to these situation or circumstances but to no others. The alternative was to renounce and waste the “opportunity” to carry out such “natural experiment”.

## Appendix: Principal component analysis

The Stata command (**factor**, **pcf**) combines the PCA and factor analysis procedure. It runs a factor analysis but rescales the estimates such that they conform to a PCA. It therefore assumes that the entire variance is common but produces (rotated) loadings, which facilitate the interpretation of the factors/component. To better interpret our factors/components, the “promax” oblique rotation technique with Kaiser criterion is used (Mooi et al., 2018).

**Table 1A.1:** Factor analysis results

Factor analysis/correlation Method: principal-component factors Rotation: oblique promax (Kaiser on)		Number of obs = 401 Retained factors = 5 Number of params = 85	
**Factor**	**Variance**	**Proportion**	**Rotated factors are correlated**
Factor1	5.01231	0.2638	
Factor2	3.73850	0.1968	
Factor3	3.55544	0.1871	
Factor4	2.30477	0.1213	
Factor5	1.66350	0.0876	

LR test: independent vs. saturated: chi2(171) = 3376.61 Prob > chi2 = 0.0000

**Table 1A.2:** Rotated factor loadings (pattern matrix) and unique variances

Variable	Factor1	Factor2	Factor3	Factor4	Factor5	Uniqueness
Job stability				0.5339		0.4786
Social prestige/career success				0.8564		0.2535
Serene family life		0.8088				0.3345
Happy love life		0.8207				0.4067
High income				0.8481		0.2932
Good health		0.5859				0.4170
Good personal relations		0.7234				0.4156
Spare time		0.4159				0.6998
Public health system			0.7272			0.3472
Pollution			0.7686			0.3173
High quality public services			0.8255			0.3026
Threatened sense of community	0.4030					0.5992
Immigration					0.9072	0.1730
Corruption	0.8849					0.2127
Crime	0.8239					0.2588
Organised crime (mafia)	0.9022					0.1738
Inequality	0.7635					0.3876
Tax evasion	0.8845					0.2589
Loss of national identity	0.4430				0.5809	0.3306

(blanks represent abs(loading) < .3)

**Table 2A:** Regression results: Proportional Odds Ratio

Variables	*Models*			
*Happiness*	(1)	(2)	(3)	(4)
Social capital	1.144**	1.153***	1.150***	1.146***
	(2.399)	(2.608)	(2.638)	(2.584)
Health and relationships	1.482***	1.474***	1.428***	1.436***
	(3.644)	(3.722)	(3.529)	(3.555)
Relational-covid	0.553***	0.547***	0.561***	0.568***
	(-2.732)	(-2.843)	(-2.705)	(-2.644)
Income-covid	0.817	0.805	0.778	
	(-0.942)	(-1.035)	(-1.237)	
Health-covid	1.064			
	(0.294)			
Public goods	0.907	0.901		
	(-1.056)	(-1.157)		
Materialism	1.191	1.163		
	(1.566)	(1.390)		
Populism	1.142	1.149		
	(1.139)	(1.205)		
Education	1.375			
	(1.550)			
Hometown population	0.646**	0.646**	0.645**	0.662**
	(-2.192)	(-2.202)	(-2.202)	(-2.122)
Age	0.997			
	(-0.406)			
Gender	0.602**	0.614**	0.651**	0.640**
	(-2.489)	(-2.418)	(-2.126)	(-2.210)
Gender# Health and relationships	0.681***	0.693***	0.727**	0.716**
	(-2.759)	(-2.647)	(-2.387)	(-2.477)
Religious faith	2.075***	1.996***	1.984***	2.008***
	(3.534)	(3.524)	(3.525)	(3.578)
Constant cut1	0.127***	0.111***	0.116***	0.130***
	(-4.809)	(-7.126)	(-6.895)	(-7.062)
Constant cut2	1.056	0.912	0.931	1.041
	(0.129)	(-0.315)	(-0.241)	(0.148)
Observations	397	397	397	397
Wald	47.12	45.01	39.20	37.46
Prob > Chi2	1.84e-05	4.84e-06	4.51e-06	3.84e-06
PseudR2	0.0657	0.0625	0.0549	0.0530
LL	-391.8	-393.2	-396.4	-397.2

**Table 3A:** Brant Test of Parallel Regression Assumption

**Variable**		***chi2***	***p > chi2***		***df***			
All		3.98	0.782		7			
Social capital		0.12	0.726		1			
Health and relationships		0.31	0.577		1			
Relational-covid		0.07	0.785		1			
Hometown population		1.46	0.227		1			
Gender		0.63	0.428		1			
Gender# Health and relationships		0.06	0.809		1			
Religious faith		1.03	0.311		1			

* A significant test statistic provides evidence that the parallel regression assumption has been violated*

*The above test shows that the proportional odds assumption has not been violated*

## Footnotes

1 It is worth noting that in 2020 the Gallup’s World Polls was forced to modify its approach to how poll the world, from face-to-face to telephone interviews.

2 Among the other, for example the European Social Survey assesses people’s happiness on a 10-point scale, ranging from extremely unhappy to extremely happy. The same is true for the Gallup World Poll. Cummins & Gullone (2000) recommend using a numerical scale to 10 to measure SWB in order to increase scale sensitivity. The same suggestion is given by the OECD in its set of guidelines to measure SWB (OECD 2013).

3 All the investigated items are presented in Table 2.

4 The score of our sample is slightly lower than those obtained by ISTAT (2019). This may depend on the particular period and situation of respondents, but also on different characteristics of the sample.

5 Eigenvalues represent the total amount of variance that can be explained by a given principal component. They can be positive or negative in theory, but in practice they explain variance which is always positive. Eigenvalues are also the sum of squared component loadings across all items for each component, which represent the amount of variance in each item that can be explained by the principal component (Jolliffe, 2002),

6 As a check we also performed factorial analysis. While the two approaches are quite similar in computational procedure and often almost similar in the results, it is well known that they are different from a conceptual point of view. In any case, we were reassured by the fact that the results were by and large the same.

7 The analysis set aside two domains: spare time and loss of sense of community. Neither seemed to have any effect on happiness.

8 This result also emerged when considering the relational covid as the unique explanatory variable, with p < 0.1.

9 This figure is calculated as ${e}^{-0.593}-1=-0.447$, where exp(-0.593) = 0.5527 is the proportional odds ratio (see Table 2 A in the appendix). It means that when the independent binary variable (social-covid) assumes 1 (much and very much) rather than 0 (very little and little), the probability of reporting higher levels of happiness decreases by about 45%, or that it is 0.5527 times lower, other variables in the model held constant. Because of the parallel regression assumption, this applies to each pair of outcome groups. This is one of the assumptions underlying OLR: the variable coefficient describing the relationship between the lowest versus all higher level of the response variable is the same as those that describe the relationship between the next lowest level and all higher levels. We tested for it and result provides evidence that the parallel regression assumption is accepted (see Table 3 A in the appendix).

10 This was also found for Scotland (Gilbert, Colley, and Roberts 2016).
